# Speech motor learning changes the neural response to both auditory and somatosensory signals

**DOI:** 10.1038/srep25926

**Published:** 2016-05-16

**Authors:** Takayuki Ito, Joshua H. Coppola, David J. Ostry

**Affiliations:** 1 Haskins Laboratories, 300 George Street, New Haven, CT, 06511, USA; 2 CNRS, GIPSA-LAB, 11 rue des Mathématiques, Grenoble Campus BP46, F-38402, Saint Martin D’heres Cedex, France; 3 Univ. Grenoble-Alpes, F-38040 Grenoble, France; 4 McGill University, 1205 Dr. Penfield Avenue, Montréal, QC, H3A 1B1, Canada

## Abstract

In the present paper, we present evidence for the idea that speech motor learning is accompanied by changes to the neural coding of both auditory and somatosensory stimuli. Participants in our experiments undergo adaptation to altered auditory feedback, an experimental model of speech motor learning which like visuo-motor adaptation in limb movement, requires that participants change their speech movements and associated somatosensory inputs to correct for systematic real-time changes to auditory feedback. We measure the sensory effects of adaptation by examining changes to auditory and somatosensory event-related responses. We find that adaptation results in progressive changes to speech acoustical outputs that serve to correct for the perturbation. We also observe changes in both auditory and somatosensory event-related responses that are correlated with the magnitude of adaptation. These results indicate that sensory change occurs in conjunction with the processes involved in speech motor adaptation.

The idea that motor learning involves changes to sensory systems has been documented previously in the context of human limb movement^1^,^2^,^3^,^4^,^5^,^6^,^7^. Sensory change has been reported using psychophysical measures^1^,^2^,^3^,^5^, evoked sensory responses^4^, and in neuroimaging studies, changes to functional connectivity in sensory networks of the brain^6^. Changes to sensory systems would also seem to be integral to speech motor learning since the acquisition and refinement of sensory targets is fundamental to the finely articulated movements of speech. There have been a number of reports using behavioral measures which indicate that there is perceptual change in association with speech motor adaptation^8^,^9^. Accordingly one would expect that adaptation would also be accompanied by changes to the neural coding of sensory inputs. Changes to the neural processing of both auditory and somatosensory inputs would be anticipated since there is correlated auditory and somatosensory feedback throughout the learning process.

There is behavioral data showing that speech motor adaptation is associated with changes to auditory and somatosensory function^10^,^11^,^12^. In these studies, sensory feedback is altered either by changing the spectral characteristics of participants’ vocal utterances and playing them back to them in real-time through headphones or alternatively by using a robotic device that alters speech articulatory movements and hence somatosensory feedback. Patterns of adaptation in response to these changes are similar to those observed in limb movement (e.g. Krakauer^13^, Shadmehr and Wise^14^), although the compensation is incomplete. When the perturbation is introduced abruptly there is a rapid change in speech motor output and associated speech sounds that serve to compensate for the alteration. When the perturbation is removed, there is a gradual return to baseline values.

Both auditory and somatosensory perturbations in speech have been observed to result in changes to measures of auditory perception^8^,^9^,^15^. In both cases, shifts in the perceptual boundary occur between the utterance involved in the sensorimotor training and other related speech sounds. In adaptation to altered auditory feedback, the perceptual shifts appear to be driven by motor outflow. The perceptual change is associated with what the participant must say in order to adapt rather than with what the participant hears^8^. In speech studies, the perceptual change is often correlated with the magnitude of learning^9^. However Lametti, *et al*.^8^ do not observe this relationship. In studies of limb motor control, perceptual change is generally observed in relation to adaptation^1^,^3^,^5^.

Behavioral observations are thus consistent with the idea that speech motor adaptation is associated with changes to sensory function. However, no direct physiological evidence of sensory change has been reported. In the present paper we use auditory and somatosensory event related potentials to test for changes to the neural processing that occur in conjunction with speech motor adaptation. For auditory analyses, we focus on electrode locations over left and right frontal cortex and along the midline. These frontal locations have been previously associated with auditory potentials^16^,^17^. For somatosensory analyses, and in particular the time-frequency analysis, we focus on activity over orofacial sensorimotor regions. Previous studies^18^,^19^,^20^ have documented mu rhythms (11–13 Hz cortical oscillations) over these sites. We find that adaptation results in changes to sensory evoked potentials. We also find that the magnitude of the electrophysiological change is correlated with behavioral measures of learning. However, the particular electrophysiological changes are different for auditory and somatosensory stimuli. Nevertheless, in each case, sensory change appears to be a part of the process of speech motor adaptation, and presumably learning.

## Results

We evaluated changes in cortical sensory processing by recording somatosensory and auditory event-related potentials in response to somatosensory stimulation (facial skin stretch) and auditory stimulation (synthesized vowel sounds). Sensory responses were recorded before and after speech motor training.

**Figure 1:**
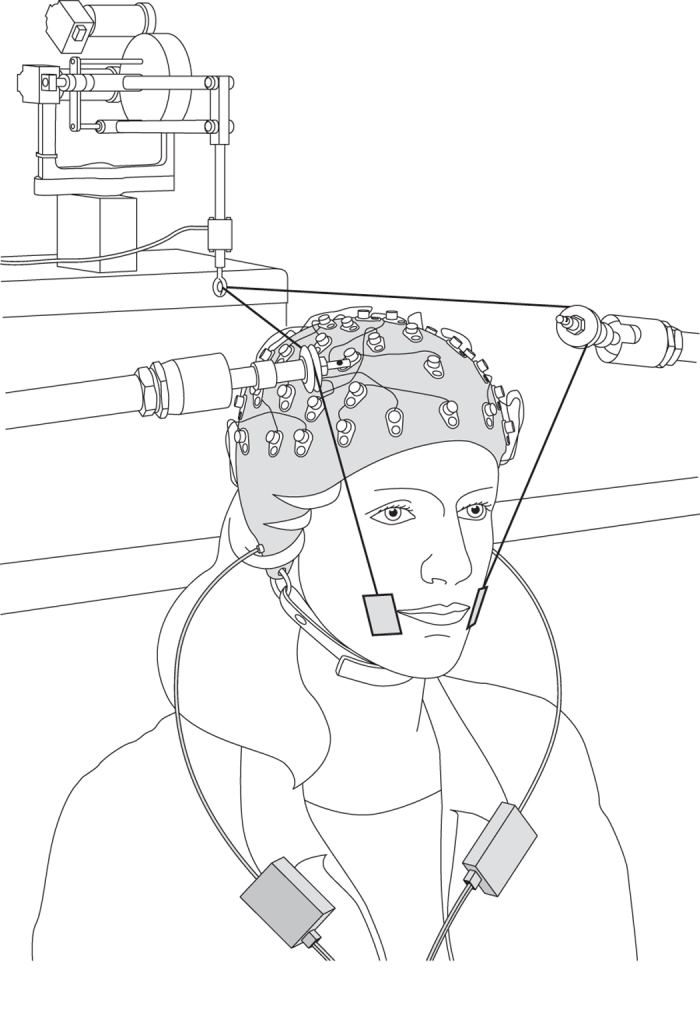
Experimental setup with 64 channel cap for recording cortical event-related potentials, somatosensory stimulation device associated with facial skin stretch, and EEG-compatible earphones.

**Figure 2:**
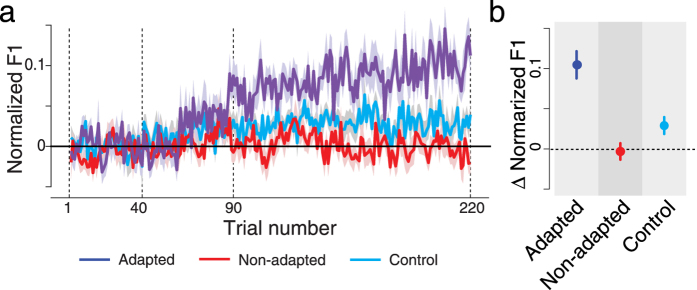
(**a**) Normalized first formant frequency over the course of training with altered auditory feedback. The shaded area represents one standard error across participants. The vertical dotted lines show the start (40) and end (90) of the ramp phase of the F1 shift. The dotted lines at 1 and 220 show the beginning and end of training. We aligned the behavioral data using the average F1 frequency between trials 21–40, which is the baseline level before the beginning of altered auditory feedback. (**b**) Average amplitude of first formant change due to speech motor training. Error bars represent one standard error across participants. The vertical axes in both panels represent normalized F1 values relative to baseline productions of F1.

We first examined the effects of altered auditory feedback on speech motor adaptation (see Fig. 1 for experimental setup). We tested 18 participants with this procedure and found that half of participants showed reliable adaptation effects. This proportion is comparable to, although slightly lower, than other reports in the literature^11^,^21^. Figure 2a shows changes to the first formant frequency in the produced vowel over the course of training. The blue line shows the data averaged across the nine individuals who adapted to the formant shift. The shaded area represents the standard error across these participants. The data are aligned based on the average F1 frequency between trials 21 and 40, which are the final base-line trials before the training phase. The alignment was carried out in order to enable a comparison of the amount of adaptation in the different groups of participants. It can be seen that the first formant in the production of /ɛ/ gradually increased over the course of the ramp phase (trials 40–90) in response to a gradual lowering of F1 in the participant’s auditory feedback. The change in F1 frequency was maintained over the remainder of the training trials. The other 9 participants in the experimental condition showed no formant shift (Red line). There was also no change of the first formant frequency in the control group (Cyan line). We calculated the average first formant frequency at the beginning and end of training and quantified the change by subtraction (Fig. 2b). The error bars show the standard error across the participants. A one-way ANOVA indicated that the pattern of F1 frequency change following training differed reliably for the three groups of participants [F(2,24) = 18.34 p < 0.001]. Pairwise comparison with Bonferroni correction showed a significant difference between adapted and non-adapted and between adapted and control groups (p < 0.002 for both comparisons), but not between the non-adapted and control participants (p > 0.25). Note that the control group on its own is marginally different from zero (p = 0.07). Accordingly, the sensory cortical response analysis that follows was conducted separately for these three groups (adapted, non-adapted and control).

Auditory and somatosensory event-related responses were recorded before and after speech motor adaptation. We applied two types of event-related analysis: potential analysis and time-frequency analysis using the Morlet wavelet transformation. We found a number of changes that were specifically related to adaptation. In particular, there was a reliable reduction of auditory event-related potentials at the first positive peak around 200 ms (P2) and a reliable enhancement of somatosensory responses between 11–13 Hz in the time-frequency analysis.

**Figure 3:**
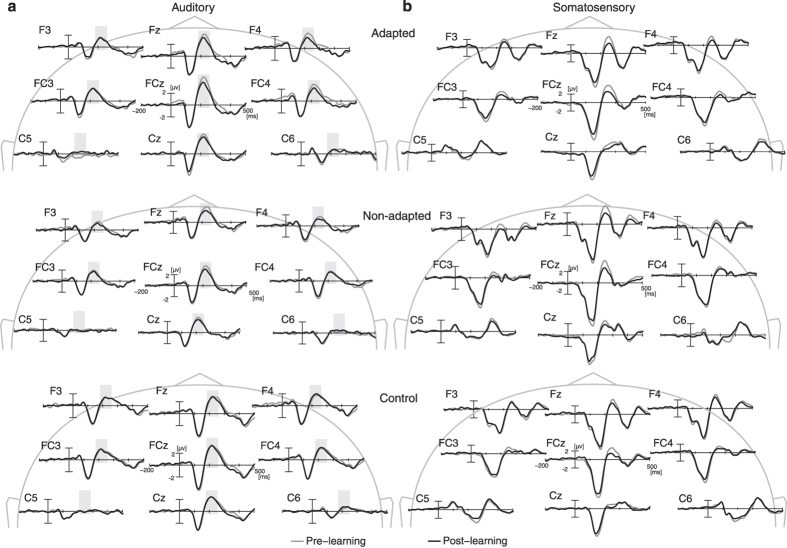
Temporal pattern of auditory (**a**) and somatosensory (**b**) event-related potentials (ERPs) at representative electrodes in frontal regions. Gray lines represent ERPs before the training. Black lines represent ERPs after the training. The shaded area shows the time-window for the auditory ERP amplitude calculation.

We first present the results of the auditory potential analysis. In all three groups of participants (adapted, non-adapted and control) we consistently obtained a typical N1-P2 sequence, that is, a first negative peak in the event related response around 100 ms after auditory onset (N1) and then a second positive peak (P2) at around 200 ms after onset. This pattern of auditory ERPs is seen at electrode locations over the frontal region. Figure 3a (left side) shows temporal patterns of auditory event-related potentials at representative frontal electrode locations (F3, Fz, F4, FC3, FCz, FC4, C5, Cz, and C6). The top panel shows the adapted participants, the middle panel shows the non-adapted participants and the bottom panel shows data for the control group. Each line represents the averaged data across participants. Black lines show the potentials in the post-training trials while gray lines indicate the potentials in the pre-training trials. The gray squares show the time bin that was used to calculate the peak amplitude of the response. The greatest amplitude potentials are at mid-line electrode locations (Fz, FCz, and Cz). The N1-P2 patterns and associated electrode locations are consistent with previous findings for auditory event-related potentials^16^.

**Figure 4:**
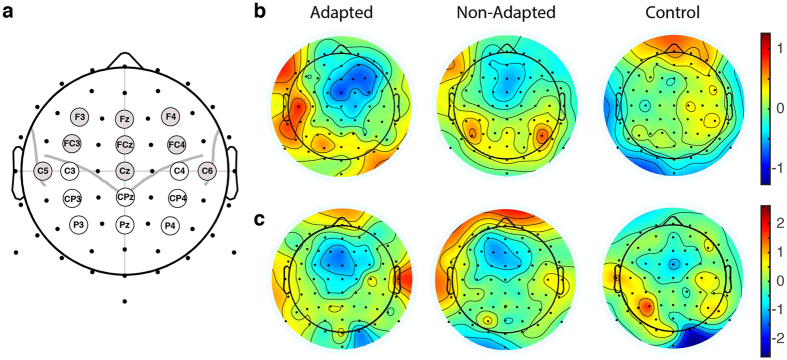
(**a**) Electrode locations for auditory statistical analysis shown in gray. (**b**,**c**) Topographic representation of differences in auditory (**b**) and somatosensory (**c**) ERPs from before to after training.

We evaluated amplitude differences in N1 and P2 potentials between pre- and post- training and found amplitude change only in P2 potentials. Figure 3a shows that, at several electrode locations, the P2 potentials are smaller after training than before training. This can also be seen in Fig. 4b, which gives a topographic mapping of differences in potential amplitude from before to after training. The adapted group shows a reduction in the P2 amplitude in electrodes over the right frontal area. The non-adapted group shows a slight reduction at electrodes along frontal midline. There were no changes observed in control group.

We assessed the differences in P2 magnitudes quantitatively using split-plot ANOVA at electrode locations in the left and right hemisphere and along the midline of the frontal region (see Fig. 4a). We focused on these locations because the auditory ERP is typically greatest in these areas of frontal cortex, in particular along the midline. Electrodes over the right hemisphere showed changes in amplitude after learning that differed in magnitude for participants in the adapted, non-adapted and control groups. This pattern was observed when activity was averaged over F4 and FC4 [F(2,24) = 5.16, p < 0.04] and also for activity averaged over F4, FC4 and C4 [F(2,24) = 5.24, p < 0.04]. A statistically significant decrease in the amplitude of event-related potential was observed in the adapted group (p < 0.005). No statistically reliable change in the amplitude of the event-related potential was observed in the non-adapted (p > 0.9) and control groups (p > 0.9). There were no learning related changes in any of the groups in event related potential amplitudes in the electrodes over the left hemisphere (averaged over F3 and FC3 or over F3, FC3 and C3) or along the midline (averaged over Fz and FCz or over Fz, FCz and Cz).

We also examined whether changes in cortical activity associated with auditory inputs were correlated with behavioral measures of adaptation to altered auditory feedback. We first conducted this analysis for adapted, non-adapted and control groups separately. In general, we found that for the adapted group, the magnitude of the P2 peak reduction in response to auditory stimuli was correlated with the magnitude of the normalized F1 change in speech adaptation trials. That is, participants that showed greater adaptation also had a greater reduction in the amplitude of the auditory evoked response. We specifically tested these correlations at frontal electrode locations above left and right hemisphere and at the midline (described above). We found reliable correlations for the adapted group, at electrodes above the right hemisphere (averaged across F4 and FC4: r(8) = −0.726, p < 0.03, averaged across F4, FC4 and C4: r(8) = −0.743, p < 0.03). The correlations for left hemisphere and midline electrodes were not reliable (p > 0.4 in all cases). The same analyses were repeated in non-adapted and control groups. There were no reliable correlations for these groups between ERP measures and the amount of adaptation.

We also assessed, for adapted and non-adapted groups together, the correlation between the magnitude of the P2 peak reduction and the magnitude of the normalized F1 change. In this case, there were reliable correlations, again over the right hemisphere electrodes between ERP reduction and amount of adaptation (averaged across F4 and FC4: r(17) = −0.65, p < 0.003, averaged across F4, FC4 and C4: r(17) = −0.69, p < 0.002). The correlations were not reliable for the left hemisphere or the midline electrodes (p > 0.15 in both).

Somatosensory event-related potentials are shown in Fig. 3b (right side). A topographic mapping of differences in potential amplitude from before to after training is shown in Fig. 4c. As is observed for auditory ERPs, the somatosensory potentials show a negative-positive peak sequence particularly around mid-sagittal electrode locations. The first negative peak is around 100 ms after stimulus onset. A second positive peak follows afterwards. Unlike auditory ERPs, we observed a reliable reduction in the peak amplitude (mostly in the second positive peak) of the post-learning response in all three groups of participants (adapted, non-adapted and control) [Left hemisphere, F3 and FC3: F(1,24) = 11.213, p < 0.01, F3, FC3 and C3: F(1,24), p < 0.02, Right hemisphere, F4 and FC4: F(1,24) = 7.191, p < 0.05, F4, FC4 and C4 : F(1,24) = 6.074, p = 0.06, Middle line, Fz and FCz: F(1,24) = 21.861 p < 0.0001, Fz, FCz and Cz: F(1,24) = 20.504 p < 0.001]. In this analysis, the reduction was non-specific in the sense that it was not related to the presence or absence of the auditory perturbation nor to the presence or absence of adaptation.

**Figure 5:**
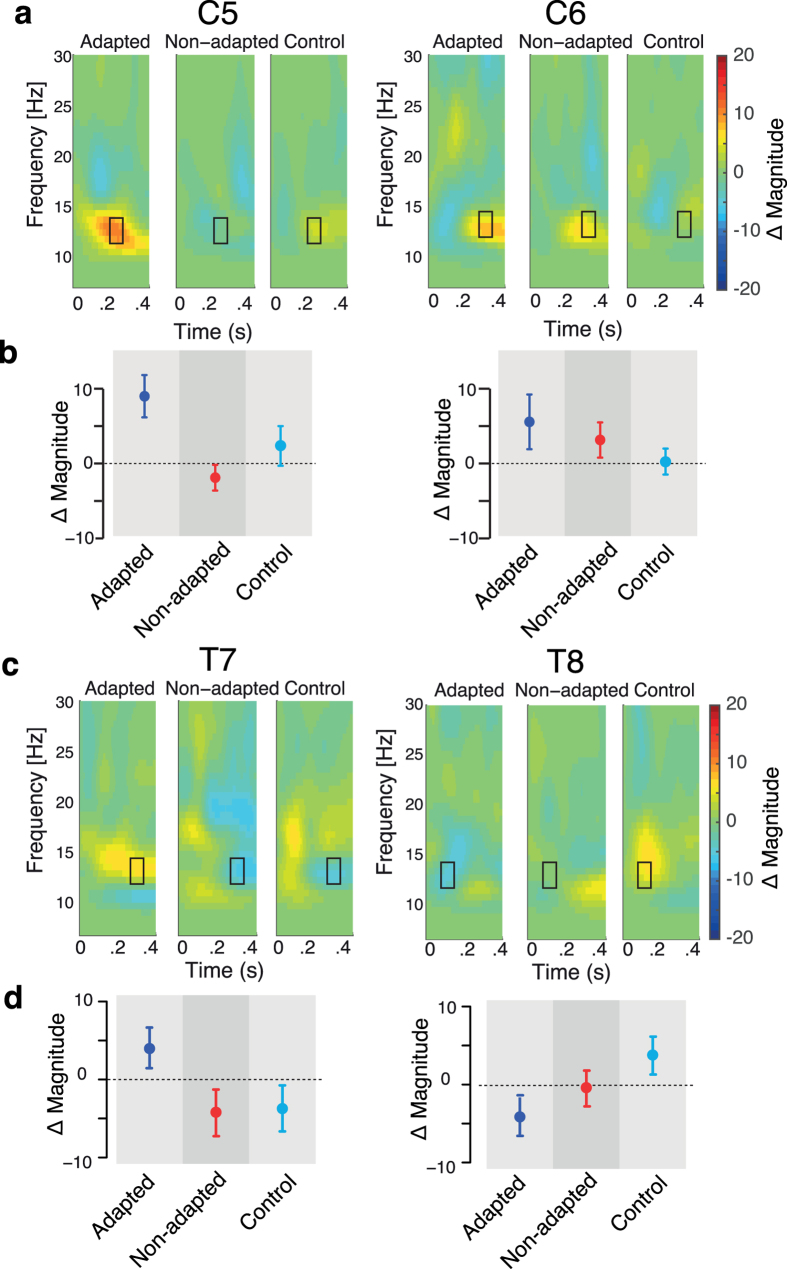
Time-frequency decomposition of somatosensory and auditory event-related responses using Morlet wavelets. (**a**) Time-frequency plot of somatosensory amplitude change between pre- and post-training over orofacial sensorimotor cortex (C5: left hemisphere and C6: right hemisphere). The black rectangle represents the time-frequency window used to calculate peak amplitude. (**b**) Average amplitudes of time-frequency somatosensory change. Error bars represent one standard error across participants. (**c**) Time-frequency plot of auditory amplitude change between pre- and post-training over auditory cortex (T7: left hemisphere and T8: right hemisphere). The black rectangle represents the time-frequency window for peak detection as in panel (**a**). (**d**) Average amplitudes of time-frequency auditory change. Error bars represent one standard error across participants.

Changes to somatosensory processing were also assessed using a time-frequency analysis that was conducted using a Morlet wavelet transformation (wavelet width = 7). We focused on electrode locations C5 and C6 since sensorimotor activity in the alpha band range, which is known as mu rhythm, occurs at these electrodes locations. We observed an amplitude increase with speech motor adaptation in the 11 to 13 Hz range at electrodes over the left sensorimotor area (C5). Figure 5a shows a representative time-frequency pattern at electrode locations C5 and C6. The figure shows differences in the event-related response amplitude between pre- and post-training trials. The three panels at each electrode location show the data for adapted, non-adapted and control participants, respectively. It can be seen that the adapted group showed an increase in amplitude in the 11–13 Hz range at a delay of between 200 and 250 ms following the somatosensory stimulus. These same patterns are not seen for non-adapted and control participants in the same time-frequency range. We quantified the magnitude of the response change using a time-frequency window. In a first analysis, we set the center of time-window to the peak location for the adapted group and used this same time-window for the other two groups (squares shown in Fig. 5a). ANOVA indicated that the difference between pre and post-training somatosensory potentials at C5 differed reliably for the three groups of participants [F(2,24) = 5.019, p < 0.03]. Post-hoc tests with Bonferroni corrections showed a reliable difference between pre and post-training amplitudes only for participants in the adapted group (p < 0.005) but not for non-adapted or control participants (p > 0.9 in both). In contrast, the corresponding electrode, C6, in the right hemisphere did not show reliable changes in the event-related response following adaptation [F(2,24) = 0.967, p > 0.7, right panels in Fig. 5a,b]. In a second analysis, we set the center of time window associated with the peak of amplitude difference separately for each group. Once again, ANOVA indicated that the difference between pre and post-training differed reliably for the three groups of participants at C5 [F(2,24) = 5.24, p < 0.03], but not at C6 [F(2,24) = 1.663, p > 0.4]. Post-hoc tests with Bonferroni corrections at C5 showed a reliable difference only for participants in the adapted group (p < 0.01) but not for non-adapted or control participants (p > 0.8 in both). This suggests that the somatosensory circuit in the left hemisphere is more closely tied to speech motor adaption with altered auditory feedback. It should be noted that in the analyses reported above there were two individual participants (one in adapted group and the other in non-adapted group) that showed extreme outlier scores in beta band range (15–25 Hz). The beta band frequencies of these participants were removed in the data presented in Fig. 5a.

We assessed whether changes in the amplitude of the somatosensory response with learning were correlated with the magnitude of the first formant change. When we carried out the correlation analyses separately for each group of participants, we did not find a reliable correlation. However, with the adapted and non-adapted groups combined, we found that, at the C5 electrode location, the change in the somatosensory response following learning was correlated with the magnitude of the normalized F1 change [r(17) = 0.56, p < 0.05 when using the same time-window, r(17) = 0.58, p < 0.05 with the time-window set separately for each group]. On the other hand, there was no reliable correlation for the responses at C6 [r(17) = 0.02 p > 0.9 for the same time-window, r(17) = 0.17 p > 0.4 with the time-window set separately for each group]. The results for somatosensory stimuli are thus restricted, both spatially and in the time-frequency domain. Nevertheless, they are consistent with the idea that speech motor adaptation likewise alters the neural processing of somatosensory inputs.

A time-frequency analysis was also carried out on the auditory potentials. We focused on electrode locations T7 and T8 as these electrodes correspond to auditory cortex (Fig. 5c). An examination of this figure suggests differences in alpha band frequency range. However, these effects were not found to be statistical significant with Bonferroni correction. We also observed modest enhancements for adapted participants in the same frequency band as in somatosensory analysis, but at different electrode locations. The change was present at electrode locations over right parietal cortex (CP2, CP4, P2 and P6). The magnitude of the change was reliable using uncorrected p-values, however, they failed to reach significance following Bonferroni correction.

The pattern of somatosensory event-related responses merits further comment. As mentioned above, there was a reduction in the magnitude of somatosensory ERPs (Fig. 3a), primarily in the first positive peak in all three groups of participants (adapted, non-adapted and control). These changes in sensory responses may possibly be related simply to word repetition in the training task, or even to stimulus repetition during the ERP recording. This may be an indication that the simple repetition task that we used as a control condition may itself entail a kind of learning associated with sensory changes.

## Discussion

Speech production training with altered auditory feedback results in adaptive changes to speech vocal output and also results in changes to cortical auditory and somatosensory processing. We found that auditory event-related potentials were reduced post-training at electrodes over right frontal regions. In somatosensory event-related responses, we found an enhancement of activity at frequencies between 11 and 13 Hz, over sensorimotor cortex. We failed to find learning related changes in somatosensory signals in an ERP analysis. The magnitude of the change in both auditory and somatosensory responses was correlated with the amount of speech motor adaptation. The results are consistent with the idea that speech motor adaptation alters the cortical processing of speech-related somatosensory and auditory inputs, and they complement a substantial literature documenting sensory plasticity produced by manipulating afferent input alone^22^.

The present findings provide physiological evidence that speech motor adaptation results in changes to neural processing in sensory systems. The finding complements previous behavioral demonstrations that adaptation to altered auditory inputs leads to changes in the perceptual classification of speech sounds^8^. The results are also consistent with a larger body of evidence from work on human limb movement^7^. Studies of arm reaching movement using both force-field adaptation and visuomotor transformations have demonstrated changes in the sensed position of the limb that occur in conjunction with learning^1^,^3^,^5^. Changes in functional connectivity in resting-state sensorimotor networks have been observed in association with the perceptual changes that occur in the context of learning^6^. There are likewise changes to short latency somatosensory potentials following force-field adaptation^4^. It is presently unknown whether learning (as opposed to adaptation) results in similar changes to sensory processing. However, the very fact that sensory change is present in conjunction with adaptation is evidence that even the rather subtle changes in motor output that are required for adaptation are sufficient to produce consistent and measurable changes to sensory function.

In the present paper, we observed that changes in both auditory and somatosensory responses were correlated with the amount of speech motor adaptation. Relationships between sensory change and adaptation have been observed previously in the behavioral studies of limb movement and speech^2^,^6^,^7^,^9^. However a number of studies that have reported both sensory changes and adaptation have failed to see a correlation^1^,^8^. The present or absence of this correlation does not appear to be related to whether visual, auditory, or somatosensory perturbations are used to provoke adaptation. The question of whether sensory change and motor adaptation magnitude are linked or independent is important in understanding the way, which sensory changes come about. While the answer remains uncertain, the physiological data reported in this paper and the behavioral results reported in the previous studies^1^,^2^,^5^,^6^,^7^,^8^,^9^ are consistent with the more general idea that aspects of sensory processing are altered in association with sensorimotor adaptation.

Auditory event-related potentials that are recorded in response to speech sounds are typically represented as an N1-P2 sequence^16^. Depending on the stimulus context, P2 morphology often varies with that of N1. Hence, N1 and P2 are usually considered to result from the same cortical mechanism. However, a recent study has shown that P2 might be independent of N1^23^. Our findings are consistent with this view, since P2 amplitude alone was altered as a result of speech motor training. While the sources of P2 variation are not well understood, the N1 component has been well investigated^17^. Previous studies of vowel processing suggest that N1 might be associated with the initial extraction of vowel related information. Edmonds, *et al*.^24^ showed a clear difference in ERP responses to noise versus vowels in the period of N1 and after. Mismatch negativity studies also find that the N1 component is sensitive to formant information related to vowel sounds^25^,^26^. In the present study, there were no changes in N1 following speech motor learning; a result that may indicate that initial processing of vowel sounds is unaltered by speech motor training. In contrast, effects are observed in the later P2 component at around 200 ms, and may reflect changes to the secondary auditory processing that arise in the context of speech motor adaptation.

We observed that speech motor adaptation is associated with changes to somatosensory event-related potentials at frequencies between 11 and 13 Hz at electrodes over left sensorimotor cortex (C5). These effects like those in auditory processing are also observed at latencies of around 200 ms. The change in the present study was observed only in individuals who showed adaptation to altered auditory feedback. More generally, in sensorimotor regions, the frequency range between 11 and 13 Hz corresponds mu rhythms that represent neural oscillations associated with somatosensory function^19^,^27^,^28^. Mu rhythms are observed in the electrodes over sensorimotor areas corresponding to leg, arm and facial motion^18^,^27^,^28^, which is consistent with our observation. Mu rhythm activity is also observed in speech production with delayed auditory feedback or with noise^20^. All of these results suggest that the mu rhythms reflect sensorimotor processing. Our results also indicate that changes in the amplitude of the mu rhythm are tied to adaptive motor responses associated with altered auditory feedback.

The two different analyses used in these studies, event-related potential (ERP) analysis and time-frequency analysis, provide tests of different cortical mechanisms. The ERP analysis extracts phase-locked activities of a large neuronal population engaged in sensory processing associated with corresponding sensory inputs^27^. On the other hand, the time-frequency analysis extracts an evoked response that is presumably related to oscillatory activity generated among multiple cortical and subcortical sites^27^. Oscillatory activities are continuously generated regardless of sensory stimulation. In the present context, the observed time-frequency responses are assumed to reflect changes to one or more parameters (areas) in the oscillatory networks for speech motor control. We observed changes associated with speech motor adaptation in both kinds of analysis. However, the relationship between the sensory oscillatory cortical network and the processing areas involved in event-related potentials is presently unknown.

There are notable similarities, but also differences, in the observed changes to auditory and somatosensory responses following adaptation. In both cases, changes are observed at latency of 200 ms or more, which suggests that the adaptation related changes are not occurring primary sensory cortices, but rather in secondary processing areas. While these results are readily understandable in the context of speech related auditory processing, the longer latency associated with somatosensory change is intriguing and might similarly suggest that changes to processing are not restricted primary somatosensory cortex. We also observed changes in auditory and somatosensory processing associated with different electrophysiological measures. The auditory changes were observed as a reduction of ERP over right frontal areas, whereas somatosensory changes occurred as an increase of alpha band activity over left somatosensory cortex. The functional significance of these specific changes needs to be established.

In summary, we have seen changes to both auditory and somatosensory potentials, which occur in conjunction with speech motor adaptation. Participants in the present study learned to produce the same vowel sound, presumably using different speech articulatory motion to correct for altered auditory feedback. In other words, participants likely had to learn to tolerate somatosensory error in order to achieve the desired acoustical outcome^29^. This remapping between speech sounds and the corresponding articulatory motions (and the associated somatosensory feedback) presumably contributes to the changes to event related cortical potentials observed here. However, the changes to neural processing appear tied to adaptation rather than altered feedback as there is altered sensory input for subjects in both the adapted and non-adapted condition. The results are thus consistent with the recent demonstration that perceptual change observed in conjunction with speech motor adaptation is related to what subjects are required to say in order to adapt, rather than with what they hear^8^ and therefore may reflect the neural processes associated with these changes in perceptual coding. As a whole, our results suggest that speech motor learning is accompanied by changes to sensory systems that occur in tandem with the changes that occur in motor systems with learning.

## Methods

### Participants and ethical approval

All experimental protocols were approved by the Yale University Human Investigation Committee, and all tests were carried out in accordance with the approved guidelines. Twenty-seven native speakers of American English participated in the experiment. The participants were all healthy young adults with normal hearing and speech. All participants signed approved informed consent forms.

### Experimental procedure

Figure 1 shows the experimental setup. We examined how speech motor training associated with adaptation to altered auditory feedback modifies auditory and somatosensory cortical processing. Over the course of training, the participants were asked to repeat aloud a task utterance. The produced vowel sounds were played back through headphones and systematically altered in real-time. In order to evaluate changes in sensory cortical processing that accompany speech motor adaptation, somatosensory and auditory event-related responses were recorded from 64 scalp sites in response to somatosensory stimulation (facial skin stretch) and auditory stimulation (synthesized vowel sounds). The event-related recording was carried out before and after speech motor training.

Eighteen participants were trained in the formant shifting condition. For comparison purposes, we carried out a control test in which the speech motor training task was carried out using unshifted speech, that is, in the absence of altered auditory feedback. Nine participants were assigned to this control condition.

### Speech motor training paradigm

In the speech motor training session we focused on changes to vowel production under conditions of altered auditory feedback. In general, vowel sounds are characterized by vocal tract resonances known as formants that differ in frequency for different vowels. The first two formants (F1 and F2) contain most of the acoustical energy and are most important in distinguishing different vowels. By changing formant frequencies in the acoustical signal, it is possible to make one vowel more or less acoustically similar to another vowel^10^,^11^. We chose the vowel /ɛ/ in “*head*” for these studies, because this sound can readily be transformed so as to sound more like /æ/ in “*had*” (by increasing the F1 frequency) or more like /I/ in “*hid*” (by decreasing the F1 frequency). This approach to formant shifting is successful in the present situation because F1 and F2 are sufficiently far apart so that manipulations of F1 do not affect F2 (see Supplementary Material for the detail of altered auditory system). In the present study, we decreased the F1 frequency so the word “*head*” sounded more like “*hid*”. The mean downward shift in F1 was 15% ± 0.5% (mean ± SE), resulting in an F1 value of 85% of the initial value for the vowel /ɛ/.

During training, the participants were asked to speak aloud the word “*head*” in response to a visual cue. The interval between visual cues was varied between 1000 ms and 1500 ms in order to avoid anticipation and habituation. The training consisted of 220 trials divided into 11 blocks of 20 trials each. During the first 40 repetitions, participants’ auditory feedback was unaltered. The first formant was then linearly shifted by a small amount per trial over the course of the next 50 repetitions. The first formant shift at the end of the ramp phase was maintained throughout the remaining 130 trials. We did not run any aftereffect trials which are carried out in most learning studies following adaptation in order to avoid washing out the training effects before testing for event-related potentials (ERPs). In a control condition, we ran exactly the same training procedure, but without formant shifts in the auditory feedback.

### EEG acquisition

ERP recording began immediately after speech motor training (and was also conducted before training). Participants were asked not to speak after training, nor did the experimenter speak to the participant. Electroencephalography (EEG) was recorded using a 64-electrode Biosemi ActiveTwo system (512 Hz sampling rate). For each participant, we recorded 200 somatosensory event-related potentials and 200 auditory event-related potentials immediately before and immediately after the speech adaptation phase of the experiment.

Somatosensory and auditory stimuli were presented in random order. We established sensory stimuli by considering the auditory and somatosensory characteristics of the adaptation task used in the speech motor training. A synthesized vowel sound /ɛ/ in “*head*” was delivered for auditory stimulation. For somatosensory stimulation, a facial skin stretch was applied in a way experienced during the production of “*head*”, as done in the previous studies^30^,^31^ (see Supplementary Material for the detail of sensory stimulation). Note that there is no electromagnetic interference with the EEG signals due to the robot used to produce the somatosensory stimulation^32^. The inter-stimulus interval was varied between 1500 and 2000 ms. The participants were asked to gaze at a fixation point (a + symbol) in order to eliminate eye movement and blinking during the EEG recording. The fixation point was removed every ten stimuli (one block). The interval between blocks was self-paced and accordingly differed over the course of the experiment and also between participants. We carried out 40 ERP blocks (200 auditory and 200 somatosensory responses) in total with the stimulation order entirely randomized.

### Data analysis

Speech motor learning was evaluated by assessing changes in the first formant frequency (F1) over the course of the speech repetitions. F1 values for /ɛ/ in repetitions of ‘*head*’ were extracted using Linear Predictive Coding analysis^33^. We expected that the F1 of the produced vowel would change in a direction opposite to that of the experimental manipulation. That is, the F1 of the produced vowel would be expected to increase as the F1 of vowel feedback signal decreased^10^,^11^,^34^. We obtained quantitative measures, by normalizing the obtained formant frequency across participants by dividing each participant’s F1 values by their average F1 frequency across trials 1–40, which preceded the introduction of auditory altered feedback. The amplitude of the adaption effect was quantified using the difference in the normalized F1 value between the last 20 base-line trials (21–40) and the last 20 trials at the end of training (201–220). For categorization purposes, we used unpaired two-tailed t-tests on a per participant basis (p < 0.05) to quantify adaptation. As in other work, several individuals in the present study did not show reliable patterns of adaptation^11^,^21^. Accordingly, we analyzed separately the data of those participants that failed to adapt. As a result, our participants were divided into three groups: adapted, non-adapted and control. Overall statistical analyses across groups (adapted, non-adapted and control) were carried out using one-way ANOVA. Pairwise comparison with Bonferroni correction followed.

Event-related response data were analyzed in two ways: potential analysis and time-frequency analysis using the Morlet wavelet transformation (see Supplementary Material for the detail of two analyses). In both analyses, we assessed changes following speech motor learning in the signal peak amplitude across the three groups of participants. In the potential analyses, we assessed activity at the electrode locations above the left and right hemispheres and along the midline between the hemispheres. In these analyses, we examined the activity averaged over adjacent electrodes [see Fig. 4a]. The time-frequency analyses were conducted using the electrode locations to the left and right of the midline.

In both cases, split-plot ANOVA with Bonferroni correction was used to assess changes following learning in the amplitude of the auditory ERPs and somatosensory wavelet dataset. These analyses were followed by further Bonferroni corrected multiple comparisons based on the total number of locations and subject groups (adapted, non-adapted and control).

We assessed the relationship between speech motor adaptation and changes in cortical activity by carrying out a correlation analysis between changes in peak amplitude in the EEG dataset and behavioral changes in conjunction with speech motor adaptation. Amplitude changes in EEG dataset were obtained by computing a difference in peak amplitude between pre- and post-training conditions. The change in the normalized F1 frequency between the beginning and the end of the training served as a measure of behavioral change for the correlation analysis.

## Additional Information

**How to cite this article**: Ito, T. *et al*. Speech motor learning changes the neural response to both auditory and somatosensory signals. *Sci. Rep.* **6**, 25926; doi: 10.1038/srep25926 (2016).

## Supplementary Material

Supplementary Information
