# Supplementary material for: Speech motor learning changes the neural response to both auditory and somatosensory signals
Source: Sci Rep. 2016 May 16;6:25926. doi: 10.1038/srep25926 (PMC4867601; doi:10.1038/srep25926)
Supplement: Supplementary Information [file srep25926-s1.doc]

**Supplementary Materials**

**Speech motor learning changes the neural response**

**to both auditory and somatosensory signals.**

Takayuki Ito ^1,2,3^, Joshua H. Coppola^1^, David J. Ostry ^1,4^

^1^ Haskins Laboratories, 300 George Street, New Haven, CT, 06511, USA

^2^ CNRS, GIPSA-LAB, 11 rue des Mathématiques, Grenoble Campus BP46, F-38402, Saint Martin D'heres Cedex, France

^3^ Univ. Grenoble-Alpes, F-38040 Grenoble.

^4^ McGill University, 1205 Dr. Penfield Avenue, Montréal, QC, H3A 1B1, Canada

**Altered auditory feedback system**

A microphone (Sennheiser, ME66) was set 15 cm from the mouth. The speaker’s voice was altered in real time and played back to participants using insert earphones (Etymotic research, ER3A) with the signal processing parameters described below. Speech-shaped masking noise was mixed with the processed signal presented to participants. The volume of the noise was the same for all the participants (70 dB through the earphones, as measured by a sound-level meter). Participants were asked to speak softly to avoid receiving auditory feedback other than through the earphones. Before running the experiment, we asked participants to confirm that the combined volume of pink noise and microphone input were loud enough so that their utterances could only be heard through their earphones. The experimenter did not indicate that the auditory feedback would be altered.

We used a previously described setup ^1,2^ in order to modify the first formant frequency alone. Briefly, the signal from the microphone was pre-amplified and then split into two paths, one for low frequency components (below 1000 Hz) and the other for frequency components above 1000 Hz. We used 1000 Hz to divide the signal because in adults, such as we have tested, the first formant of the target vowel /ε/ is generally below 1000 Hz and the second is generally above ^3^. In the low-frequency path, an electronic speech processor (TC Helicon, VoiceOne) first shifted all formant frequencies of the original signal. The speech processor produced formant shifts on 0-50 scale relative to base line values. In the present experiment, we used the maximum shift. This results in changes in formant frequency that differ between subjects. The average F1 change in the current study was approximately 15 %, which is similar to the previous studies ^1,2^. After being shifted, the signal was analog low-pass filtered with a cut-off frequency of 1000 Hz so as to extract the frequency component in the range of the first-formant while discarding all higher frequencies. In the high-frequency path, the signal was electronically delayed by 11 ms. This delay compensated for an equal delay introduced by the formant processor and the filter in the low-frequency path. This signal was then analog high-pass filtered at the same frequency as that used in the low-frequency path. This process excluded frequency components in the range of the first formant and below but preserved the unshifted higher formant frequencies. The signals from the two paths were then mixed and the resulting signal was amplified and played back to participants through headphones. Note again that this manipulation affected only formant frequency. The remaining acoustic parameters (pitch, duration, and tone) were the same as that in the original speech output. The speech sound that the participant produced and the one which the participant heard through the headphones (the sound that included the processed formant shifts) were digitally sampled at 44,100 Hz.

**Sensory stimulation**

For purposes of auditory stimulation, a synthesized vowel sound was delivered binaurally through plastic tubes and earpieces (Etymotic research, ER3A). We used the vowel /ɛ/ in “*head*”, because it was also used as the target vowel in the speech motor training session. The stimulus sound was generated using a Klatt synthesizer ^4^. The first and second formant values of /ɛ/ were set 537 Hz and 1640 Hz, respectively, based on average values across 5 tokens of *head* provided by a male native speaker of English. The stimulus duration was 333 ms which is comparable to the duration for the production of /ɛ/ in “*head*”. At the beginning of each experimental session, we verified that participants were able to recognize the synthesized vowel as /ɛ/ without any pre-instruction.

For somatosensory stimulation, we programmed a small robotic device to apply skin stretch loads to evoke somatosensory event-related responses (see Figure 1). The details of the somatosensory stimulation procedure are described in our previous studies ^5,6^. Briefly, the skin stretch was produced by using two small plastic tabs that were attached bilaterally with tape to the skin at the sides of the mouth. The skin stretch was applied upward. We applied a single cycle of a 3-Hz sinusoidal pattern with 4 N maximum force. This temporal pattern of facial skin stretch is similar to that in the production of ‘*head*’. This same pattern of facial skin stimulation has been used to induce somatosensory event-related potentials in a previous study ^7,8^.

**Event-related response analyses**

In the potential analysis, EEG signals were filtered using a 1-30 Hz band-pass filter and re-referenced to the average across all electrodes. Bias levels were adjusted using the average amplitude in the pre-stimulus interval (-200 to -100 ms). Trials with blinks and eye movement were eliminated offline on the basis of horizontal and vertical electro-oculography (over +/- 150 mV). More than 85% of trials per condition were included in the analysis. For each participant, ERPs were averaged across trials. In the analysis of the auditory ERP, we focused on the interval associated with the first negative peak (N1) and the first positive peak (P2). For the somatosensory ERP, we focused on the peak response in two intervals (100-200 ms and 200-300ms after stimulus onset), based on our empirical observations that the overall temporal pattern of somatosensory ERPs was similar to that of auditory ERPs. We used a 60-ms time window about the peak to compute a measure of amplitude (see gray area in Figure 3).

In the time-frequency analysis, EEG signals were filtered using a 10-110 Hz band-pass filter and, as in the potential analyses, re-referenced to the average across all electrodes. Bias levels were adjusted using the average amplitude in the pre-stimulus interval (-200 to -100 ms). We rejected the trials with blinks and eye movement as above. The data were aligned at stimulus onset. We calculated the average potential across the trials for each individual and applied a Morlet wavelet transformation (wavelet width = 7)^9,10^ to the averaged data set. Baseline amplitudes in the interval between -50 to 0 ms were subtracted from the wavelet transformed dataset. The difference between pre- and post-training was calculated by subtracting pre-training from post-training Wavelet transformed measures. Peak amplitudes were calculated using a 60 ms, 2 Hz window (see rectangle in Figure 5).

**Reference**

1 Lametti, D. R., Nasir, S. M. & Ostry, D. J. Sensory preference in speech production revealed by simultaneous alteration of auditory and somatosensory feedback. *J Neurosci* **32**, 9351-9358, doi:10.1523/JNEUROSCI.0404-12.2012 (2012).

2 Rochet-Capellan, A. & Ostry, D. J. Simultaneous acquisition of multiple auditory-motor transformations in speech. *J Neurosci* **31**, 2657-2662, doi:10.1523/JNEUROSCI.6020-10.2011 (2011).

3 Hillenbrand, J., Getty, L. A., Clark, M. J. & Wheeler, K. Acoustic characteristics of American English vowels. *J Acoust Soc Am* **97**, 3099-3111 (1995).

4 Klatt, D. H. Software for a cascade/parallel formant synthesizer. *J Acoust Soc Am* **67**, 971-995 (1980).

5 Ito, T. & Ostry, D. J. Somatosensory contribution to motor learning due to facial skin deformation. *J Neurophysiol* **104**, 1230-1238, doi:10.1152/jn.00199.2010 (2010).

6 Ito, T., Tiede, M. & Ostry, D. J. Somatosensory function in speech perception. *Proc Natl Acad Sci U S A* **106**, 1245-1248, doi:10.1073/pnas.0810063106 (2009).

7 Ito, T., Johns, A. R. & Ostry, D. J. Left lateralized Enhancement of orofacioal somtosensory processing due to speech sounds. *Journal of Speech Language and Hearing reserarch* **56**, 1875-1881 (2013).

8 Ito, T., Gracco, V. L. & Ostry, D. J. Temporal factors affecting somatosensory-auditory interactions in speech processing. *Frontiers in psychology* **5**, 1198, doi:10.3389/fpsyg.2014.01198 (2014).

9 Oostenveld, R., Fries, P., Maris, E. & Schoffelen, J. M. FieldTrip: Open source software for advanced analysis of MEG, EEG, and invasive electrophysiological data. *Computational intelligence and neuroscience* **2011**, 156869, doi:10.1155/2011/156869 (2011).

10 Tallon-Baudry, C. & Bertrand, O. Oscillatory gamma activity in humans and its role in object representation. *Trends Cogn Sci* **3**, 151-162 (1999).
